# Ketoacidosis in a non-diabetic woman who was fasting during lactation

**DOI:** 10.1186/s12937-015-0076-2

**Published:** 2015-11-04

**Authors:** Sarah K. Hudak, Dietrich Overkamp, Robert Wagner, Hans-Ulrich Häring, Martin Heni

**Affiliations:** 1 Department of Internal Medicine, Division of Endocrinology, Diabetology, Angiology, Nephrology and Clinical Chemistry, Eberhard Karls University Tübingen, Otfried-Müller-Str. 10, 72076 Tübingen, Germany; 2 Institute for Diabetes Research and Metabolic Diseases of the Helmholtz Center Munich at the University of Tübingen, Otfried-Müller-Str. 10, 72076 Tübingen, Germany; 3 German Center for Diabetes Research (DZD e.V.), Otfried-Müller-Str. 10, 72076 Tübingen, Germany

## Abstract

Ketoacidosis is a potential complication of type 1 diabetes. Severe ketoacidosis with a blood pH below 7.0 is only rarely seen in other diseases.

Three weeks after delivery, a young woman was admitted because of tachypnoe and tachycardia. Blood gas analysis showed a severe metabolic acidosis with a high anion gap. Further workup revealed the presence of ketone bodies in the urine with normal blood glucose and no history of diabetes. The patient reported that she had not eaten for days because of abdominal pain. After initial treatment in the ICU and immediate re-feeding, the patient’s condition rapidly improved.

While under normal circumstances fasting causes at most only mild acidosis, it can be dangerous during lactation. Prolonged fasting in combination with different forms of stress puts breast feeding women at risk for starvation ketoacidosis and should therefore be avoided.

## Background

Severe acidosis is a potentially life-threatening condition. In case of metabolic acidosis, determination of the serum anion gap helps to narrow down the differential diagnosis. An increased anion gap indicates the presence of an unusual amount of an acid that is most commonly found in ketoacidosis, lactic acidosis, renal insufficiency, and intoxications while other causes are rare.

**Fig. 1:**
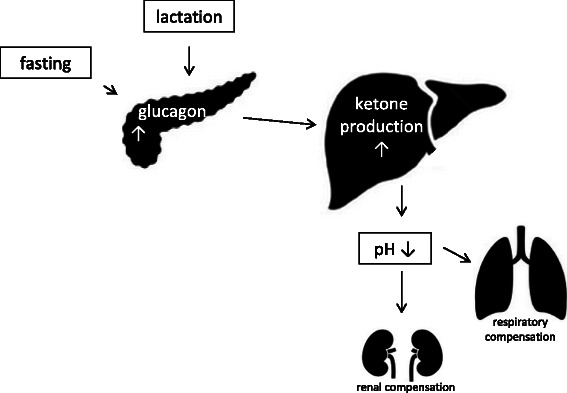
schematic overview of the pathophysiology of ketoacidosis in lactation

Ketoacidosis is a potential complication of type 1 diabetes while severe ketoacidosis with a blood pH below 7.0 is only rarely seen in other diseases. In diabetic ketoacidosis, glucose is not properly taken up into tissue due to an absolute insulin deficiency that is mainly found in type 1 diabetes. In parallel, glucagon release is not suppressed leading to hyperglucagonemia. Subsequently the body activates stress hormones, which worsen hyperglycemia by promoting gluconeogenesis (and also ketogenesis) in the liver [1]. Absence of insulin together with the other humoral derangements causes enhanced lipolysis from adipose tissue leading to a massive rise in circulating fatty acids. These fatty acids are subsequently taken up by hepatocytes. Under low insulin and high glucagon conditions, substantial amounts of these fatty acids ultimately go into ketogenesis and are then released as ketone bodies [1] (see Fig. 1). Recent research suggests, that impaired removal of ketone bodies by the kidneys and the brain also contributes to acidemia in diabetic ketoacidosis [2].

We report on a young woman who showed a severe metabolic acidosis with a high anion gap three weeks after delivery despite normal blood glucose and no history of diabetes. She was fasting while breast feeding due to constant abdominal pain.

## Case presentation

Three weeks after delivery, a 32-year-old woman was admitted to our hospital because of tachypnoea and tachycardia. She was first presented to the gynaecological unit with constant abdominal pain since delivery. The birth had been experienced as traumatic by the patient, but reports documented no complications during the vaginal delivery. The newborn daughter was the first child of the patient and was healthy. There were no abortions in the past. The patient reported not to have eaten solid food for days because of nausea and vomiting, while fluid intake caused no problems. Micturation and defecation where normal, she had no fever. Her medical history included multiple deep vein thromboses in the past (seven, five, and two years ago). Therefore, she received prophylactic treatment with low molecular weight heparin during pregnancy and after delivery. A thrombophilia screening was not yet performed because the patient rejected it for personal reasons. There where no other relevant diseases in the past history and no relevant family history.

On admission, clinical examination revealed a muscular defense over the whole abdomen and tenderness in the lower left quadrant. Gynaecological physical examination was without pathological findings. Transvaginal ultrasound revealed an ovarian venous thrombosis on the left side which was confirmed by CT-scan. No additional locations of thrombosis were detected. The dose of low molecular weight heparin therapy was increased from a prophylactic to a therapeutic regiment.

Over the next hours, tachypnoea worsened while the oxygen saturation remained normal. Increasing tachypnoea, tachycardia and hyperventilation led to the referral of the patient to the internal medicine unit.

After arrival in Internal Medicine, an arterial blood gas analysis was performed and disclosed a severe metabolic acidosis with partial respiratory compensation and a high anion gap (see Table Table 1). In addition, marked hypophosphatemia was detected. Further workup revealed the presence of ketone bodies in the urine despite normal blood glucose. She did not have a history of diabetes, and did not drink alcohol. She did not take medication regularly except prophylactic heparin during pregnancy. The presence of keton bodies might – in addition to the ovarian venous thrombosis – have also contributed to the patient’s complaints since abdominal pain is sometimes present in ketoacidosis.

**Table 1:** Laboratory measurements

	Admission to Internal Medicine	Day of admission - 6 h after initiation of treatment	Day 1	Day 2	Day 6	Reference range
	Arterial blood	Arterial blood	Arterial blood	Venous blood	Venous blood	
pH	**6.99**	**7.31**	7.44	7.45	7.40	7.35 – 7.45
pO_2_ (mmHg)	**134**	**104**	81			75 – 100
pCO_2_ (mmHg)	**8**	**11.5**	**26**			33 – 45
HCO_3_^−^ (mmol/l)	**3**	**10.5**	**20.4**	25.2	23.3	21 – 26
Anion gap (mmol/l)	**28**	**26.8**	12.7	10.5	14	8 – 16
Lactate (mmol/l)	1.0	0.8	1.7	1.3		0.5 – 2.2
Glucose (mmol/l)	**3.8**	4.5	**8.1**	**6.8**	4.4	3.9 – 6.1
Hemoglobin (g/dl)	14.5		12.0	12.1	12.8	12.0 – 16.0
White-cell count (1/μl)	10,200		10,250	6,630	11,430	4100 – 11800
Platelet count (1000/μl)	383		287	266	318	150 – 450
Sodium (mmol/l)	140	145	145	143	147	136 – 148
Potassium (mmol/l)	4.2	**3.3**	**3.2**	3.5	3.8	3.5 – 4.8
Calcium (mmol/l)	2.1	2.1		2.3	2.3	2.1 – 2.6
Phosphate (mmol/l)		**0.4**	**0.3**	**0.4**	**0.9**	0.8 – 1.5
Plasma creatinine (mg/dl)	**1.0**		0.8	0.7	0.7	0.5 – 0.8
Drug screening	Negative		Negative			Negative
Urine glucose, spot urine	Negative		Negative		Negative	Negative
Urine acetone, spot urine	**+++**		**+++**		Negative	Negative
Urine Phosphat (24-h urine collection, mmol/l)				**<1**	13	7–25

Numbers in bold lie outside of reference range

Our patient was immediately transferred to ICU and an infusion of sodium bicarbonate (8.4 %), potassium-phosphate (50 mmol/50 ml), glucose (20 %), and saline was initiated via a central line. After these measures as well as after immediate re-feeding and discontinuation of breastfeeding, acid–base homeostasis started to normalize and tachypnoea subsided. After ten days, the patient had fully recovered and was discharged home.

## Conclusions

Since we ruled out all other common forms of increased anion gap metabolic acidosis (Table 1) and because of the rapid response to re-feeding, we diagnosed starvation ketoacidosis in lactation. While under normal circumstances fasting causes at most only mild acidosis, it can be dangerous during lactation. The increased energy demand for milk production causes enhanced gluconeogenesis, decreased insulin secretion, lipolysis, and can subsequently induce ketogenesis [3]. Some reports termed this condition “bovine ketosis” [4, 5] since it is also common in dairy cows that can sometimes not compensate for the high energy requirements of milk production by sufficient energy intake [6]. In humans, this condition is rare; we are aware of only five cases in the literature [4, 5, 7–9]. Just as in our patient who suffered from abdominal pain, there was an additional stressor present in the other reported patients. Thus, prolonged fasting in combination with different forms of stress seems to put breast feeding women at risk for starvation ketoacidosis.

Given the potential harms of this life-threatening condition, complete fasting should be avoided during lactation. If fasting is necessary for medical reasons, sufficient glucose should be administered parenterally. If a patient develops starvation ketoacidosis, glucose should be administered immediately and the woman should stop breast feeding.

### Consent

Written informed consent was obtained from the patient for publication of this Case Report.
